# Account of Diseases in an Eastern District from, the 20th of December, 1799, to the 20th of January, 1800

**Published:** 1800-02

**Authors:** 


*Account of Diſeaſes in an Eaſtern Diſtrict from, the 20th of December, 1799, to the 20th of January, 1800.*

| | No. of Caſes. |
|---|---|
| ACUTE DISEASES. | |
| Typhus Mitior | 6 |
| Pneumonic Inflammation | 4 |
| Catarrh | 10 |
| Peripneumonia notha | 7 |
| Acute Rheumatiſm | 4 |
| CHRONIC DISEASES. | |
| Cough with Diſpnœa | 20 |
| Cough | 16 |
| Hæmoptoe | 3 |
| Pleurodyne | 2 |
| Phthiſis Pulmonalis | 5 |
| Hydrothorax | 4 |
| Gaſtrodynia | 5 |
| Dyſpepſia | 7 |
| Anorexia | 2 |
| Diarrhœa | 5 |
| Dyſentery | 2 |
| Enterodynia | 3 |
| Hæmorrhois | 2 |
| Procidentia Ani | 1 |
| Abortus | 1 |
| Menorrhagia | 2 |
| Amenorrhea | 3 |
| Fluor Albus | 3 |
| Gravel | 1 |
| Icterus | 1 |
| Dropſy | 3 |
| Anaſarca | 4 |
| Hemiplegia | 1 |
| Epilepſy | 1 |
| Hyſteria | 2 |
| Chronic Rheumatiſm | 13 |
| Scrophula | 4 |
| Prurigo | 2 |
| PUERPERAL DISEASES. | |
| Milk Fever | 2 |
| Ephemera | 3 |
| Dolores poſt partum | 5 |
| INFANTILE DISEASES. | |
| Meaſles | 4 |
| Hooping Cough | 2 |
| Ophthalmia | 2 |
| Dentition | 2 |
| Convulſio | 1 |
| Vermes | 2 |

A conſiderable proportion of the diſeaſes contained in the foregoing liſt had their ſeat in the cheſt; and, as it might well be expected, their number was increaſed, and the ſymptoms of them were aggravated by the ſeverity of the weather. The degree of cold, though not ſo intenſe, nor of ſo long duration, as in the laſt winter, yet gave riſe to a great number of theſe complaints. Cough, Dyſpnœa, Catarrh, and Peripneumony have been attended with very ſevere ſymptoms, and have proved, in many inſtances, unuſually tedious and obſtinate. Peripneumonia notha has very frequently occurred in perſons far advanced in life, and in a number of caſes has proved fatal.

The ſymptoms of Phthiſis Pulmonalis have alſo been much aggravated, and have advanced ſpeedily to a fatal termination.

The meaſles have lately been very prevalent, and have proved fatal in a number of inſtances. It appears by the weekly bills of mortality, that a large number has died of this diſeaſe during the months of November and December, a number amounting to more than one half of deaths, by this diſeaſe, during the whole year.

The Small-pox has proved a leſs fatal diſeaſe during the laſt year than in the preceding one, in which 2237 are ſtated in the general bill of mortality, as the number of deaths; whereas, in the laſt year, they amounted only to 111. By recurring to a monthly regiſter, we find that in January, February, November, and December, two thirds of the whole number fell a ſacrifice to this diſeaſe.

